# Development and validation of a risk prediction model for prostate cancer integrating quantitative MRI and clinical data

**DOI:** 10.3389/fonc.2026.1743678

**Published:** 2026-04-01

**Authors:** Yafei Zhang, Congyan Yin, Guochao Li, Xuhong Pan, Guowei Zhang, Ranran Huang

**Affiliations:** Department of Radiology, Yantaishan Hospital, Yantai, Shandong, China

**Keywords:** magnetic resonance imaging, multiparametric MRI, prostate cancer, quantitative analysis, quantitative imaging sequences

## Abstract

**Objectives:**

To develop and validate a predictive model for prostate cancer (PCa)by integrating quantitative MRI and clinical data.

**Methods:**

A retrospective study was conducted on 290 patients clinically diagnosed with prostate lesions between March 2023 and October 2024. All patients underwent multimodal quantitative MRI preoperatively, including T2-weighted imaging (T2WI), T1-weighted imaging (T1WI), diffusion-weighted imaging (DWI, b=0, 500 mm^2/s^), synthetic relaxometry (MAGiC), iterative decomposition of water and fat with echo asymmetry and least-squares estimation quantitation (IDEAL-IQ), and amide proton transfer (APT) sequences. Based on pathological results, patients were stratified into PCa group (n=140) and Non-PCa group (n=150). All quantitative parameters derived from multimodal MRI were measured independently by a dedicated team of radiologists, and their reproducibility was assessed through intra−class correlation coefficients (ICCs). Differences in all MRI quantitative parameters and clinical data between the groups were compared. All patients were randomly divided into a training group (203 cases) and a validation group (87 cases) at a ratio of 7:3. The Least Absolute Shrinkage and Selection Operator (LASSO) and Logistic regression were used to screen independent predictive parameters for PCa, constructing a predictive model, internal validation and bootstrap validation were performed on the model. The model was evaluated using receiver operating characteristic curves (ROC), goodness-of-fit tests (Hosmer-Lemeshow statistic), calibration curves, and decision curve analysis (DCA), and presented in the form of nomograms.

**Results:**

1) Two radiologists showed good consistency (ICCs > 0.75). 2) There were significant differences between the PCa and non-PCa groups for all variables except age(*P* < 0.05). 3) Four independent predictors were selected through LASSO-penalized logistic regression to construct the model: the Apparent Diffusion Coefficient (ADC) from DWI, T2 from MAGiC, MTRasym(3.5ppm) from APT, and Prostate-Specific Antigen (PSA). The model exhibited excellent performance, achieving an AUC of 0.986 (95% CI: 0.944-0.993); sensitivity: 92.3%; specificity: 93.3%; the optimism−adjusted area under the curve was 0.988 (bootstrap 95 % CI: 0.986–0.991), satisfactory calibration (Hosmer-Lemeshow test, *P* > 0.05), and a substantial net benefit on decision curve analysis.

**Conclusion:**

The prediction model based on quantitative MRI and clinical parameters has good performance in discrimination, stability, consistency and clinical applicability. Compared to existing diagnostic models, it offers distinct advantages in data objectivity, reproducibility, non-invasiveness, cost-effectiveness, and operational simplicity.

## 1 Introduction

Driven by economic development and improved healthcare, the incidence and detection rates of prostate cancer (PCa) have risen significantly (1). Concurrently, the disease is showing a trend toward affecting younger populations, who often present with more aggressive disease and thus may have a potentially worse prognosis (2). Precise PCa diagnosis is thus critical for early intervention. Magnetic resonance imaging (MRI) is recognized as the optimal imaging technique for the diagnosis and differential diagnosis of prostate diseases, and multiparametric MRI (mp-MRI) has been widely applied in the evaluation and diagnosis of prostate cancer (3, 4). Although mp-MRI guided by Prostate Imaging Reporting and Data System (PI-RADS) is a cornerstone of prostate cancer diagnosis, its qualitative nature remains a major limitation. Quantitative MRI (QMR) techniques overcome this limitation by providing objective tissue parameter measurements derived from MR physics principles (5). QMRI techniques such as diffusion-weighted imaging (DWI) and dynamic contrast-enhanced MRI (DCE-MRI) have been maturely applied in the diagnosis and research of PCa. However, PCa and Benign Prostatic Hyperplasia (BPH) show overlapping manifestations in DWI and DCE-MRI imaging as well as quantitative indicators, and relevant studies have shown that the rates of missed diagnosis and misdiagnosis are relatively high (6). In recent years, several emerging QMRI technologies, including magnetic resonance image compilation (MAGiC), iterative decomposition of water and fat with echo asymmetry and least-squares estimation quantitation (IDEAL-IQ), and amide proton transfer (APT), can non-invasively quantify macromolecule/polypeptide levels in PCa tissues (7–10).

MAGiC relies on quantitative information about proton density and relaxation rates to generate contrast images, producing five quantitative relaxation maps in a single imaging session: T1 mapping, T2 mapping, PD mapping, R1 mapping, and R2 mapping (11). Moreover, multiple obtained relaxation quantifications are correlated with various histopathological changes (12). Song et al. (7) found using MAGiC technology that the quantitative T1 and T2 values obtained for PCa can distinguish prostate cancer from other benign lesions, with T2 values having diagnostic performance comparable to ADC values; Hepp et al. (8) conducted research based on quantitative T2/ADC values and found that T2 mapping has high diagnostic accuracy in evaluating PCa and chronic prostatitis, with performance comparable to ADC values. IDEAL-IQ can non-invasively and quickly assess tissue fat content and paramagnetic substance content through quantitative parameters such as fat fraction (FF) values, apparent relaxation rate (R2*) values, and transverse relaxation time (T2*) values (13). Ren et al. (9) acquired high-quality 3D fat fraction maps using IDEAL-IQ technology and found that the FF value of PCa is higher than that of BPH, and its diagnostic performance is the highest compared to other parameter values, which is consistent with the abnormal lipid metabolism in PCa, and their abnormal accumulation is related to tumor progression (14). APT can non-invasively and quantitatively evaluate changes in tissue protein content and pH by detecting the chemical exchange rate between amide protons (-NH, 3.5 ppm) on free proteins and polypeptide chains and hydrogen protons in free water; the exchange rate itself is proportional to pH and is commonly quantified using the MTRasym index (15). Qin et al. (10) found that APT has a complementary role to ADC in sensitivity and specificity for distinguishing PCa with different pathological grades. In summary, the new QMRI techniques can evaluate changes in the physiological status of tumor cells at the molecular level without the need for contrast agent injection, showing good application potential and bringing more possibilities to treatment planning and prognosis assessment.

Previous studies have mostly been based on a single QMRI technique, which has limited diagnostic value. In addition, current prostate MRI examinations, due to the large number of sequences involved, result in excessively long scanning times and high costs, restricting further improvement of their diagnostic efficacy. To address this, this study aims to integrate multiple quantitative MRI techniques—including DWI, MAGiC, IDEAL-IQ, and APT—and combine them with clinical data such as age and prostate-specific antigen (PSA) to construct a new prostate cancer prediction model. We expect this model to effectively improve diagnostic efficiency and provide more valuable objective evidence for clinical decision-making.

## 2 Materials and methods

### 2.1 Subjects

This single−center retrospective study included 290 patients who were clinically diagnosed with prostate lesions between March 2023 and October 2024. According to pathological findings, patients were classified into a prostate cancer PCa group (n=140) and a non−PCa group (n=150). Clinically significant prostate cancer was defined as Gleason score ≥3 + 4; Gleason score 6 was classified as indolent prostate cancer. All pathological diagnoses were based on either biopsy or radical prostatectomy specimens; when both specimen types were available for a given patient, the prostatectomy result was used as the reference standard. All patients underwent multimodal quantitative MRI (QMRI) within one month prior to surgery, and basic clinical data, including age and prostate−specific antigen (PSA), were collected.

Inclusion Criteria: 1) Pathological confirmation via radical prostatectomy or ultrasound-guided biopsy within 1 month after MRI examination; 2) No targeted therapy was administered for prostate lesions (e.g., radiation therapy, hormone therapy) prior to the QMRI examination; 3) Availability of PSA test results within 2 weeks before/after imaging.

Exclusion Criteria: 1) Incomplete clinical documentation; 2) Suboptimal image quality precluding diagnostic evaluation.

All participants provided written informed consent, and the study protocol was approved by the Institutional Review Board and Hospital Ethics Committee (Approval No. LL-2024-127-L) in compliance with the Declaration of Helsinki.

### 2.2 MRI acquisition

The scans were performed using a 3.0T MRI scanner (SIGNA Architect, GE Healthcare) equipped with an AIR™ 40-channel torso coil. The protocol included: T2WI (T2-weighted imaging), T1WI (T1-weighted imaging), DWI, MAGiC, IDEAL-IQ, and APT sequences. Detailed imaging parameters are provided in **Table 1**.

**Table 1:** MRI acquisition parameters.

Parameter	T_1_WI	T_2_WI	DWI^†^	Synthetic MRI(MAGiC) ^∡^	IDEAL-IQ^‡^	APT^§^
TR/TE (ms)	600/9	4700/90	4000/70	4000/21	6/min	3000/min
Slice/Gap (mm)	3/1	3/1	3/1	3/1	3/1	3/1
FOV (mm²)	370x370	240×240	240x240	370x370	370x370	240×240
Time (min:s)	1:31	2:36	1:44	3:28	0:22	2:36

†DWI, b-values=0,1500 s/mm²; GRAPPA = 2; ‡IDEAL-IQ, 6-echo (TE = 1.1-7.1ms), FF/R2* mapping; §APT, B1 = 2μT, saturation=500ms, offsets= ± 3.5ppm; ∡Effective TE for multi-parametric synthesis.

### 2.3 QMRI image processing

QMRI images analysis were performed on GE Advantage Workstation 4.7. MR image interpretation and quantitative measurements were conducted by a specialist radiology team, two radiologists (one mid-career, one associate senior) independently drew regions of interest (ROI) according to the following protocols (1): MR image interpretation was performed with reference to T1−weighted and T2−weighted sequences, corresponding pathological slides, and the documented biopsy locations, the axial slice with maximal lesion cross-sectional area was selected; in multifocal cases, the dominant lesion was targeted. Identically sized ROIs were placed at consistent lesion locations across sequences, with triplicate measurements averaged as final values (2). ROIs were circular/oval, covering 50-66% of total lesion area while avoiding urethra, vascular structures, hemorrhage, calcifications, cysts, abscesses, and glandular junctions. Quantitative measurements included: ADC (DWI), T1/T2/PD (MAGiC), FF/R2^*^ (IDEAL-IQ), and MTR_asym_(3.5ppm) (APT).

### 2.4 Statistical analysis

This study is a single−center retrospective model development study that used a 7:3 random split for internal validation and applied bootstrap optimism correction, corresponding to TRIPOD Type 2b.

Statistical analyses were performed using MedCalc 20.0 (MedCalc Software Ltd) and R version 4.2.3 (R Foundation for Statistical Computing). A two-sided P-value <0.05 was considered statistically significant. Inter-rater reliability between two physicians was assessed using the intraclass correlation coefficient (ICCs).

All QMRI parameters and clinical variables underwent normality testing via the Shapiro-Wilk method. APT exhibited a right-skewed distribution and was natural log-transformed to improve symmetry. All continuous predictors were then entered into subsequent analyses on their transformed scale. Normally distributed continuous data were compared with the independent samples t-test and reported as mean ± standard deviation. Non-normally distributed data were analyzed with the Mann-Whitney U test and expressed as median (25th percentile, 75th percentile) [M(P25, P75)].Categorical variables were evaluated by Chi-square test.

Predictor selection was conducted in R using: Least Absolute Shrinkage and Selection Operator (LASSO) regression for preliminary screening. Multivariable binary Logistic regression to identify independent predictors.

**Figure 1:**
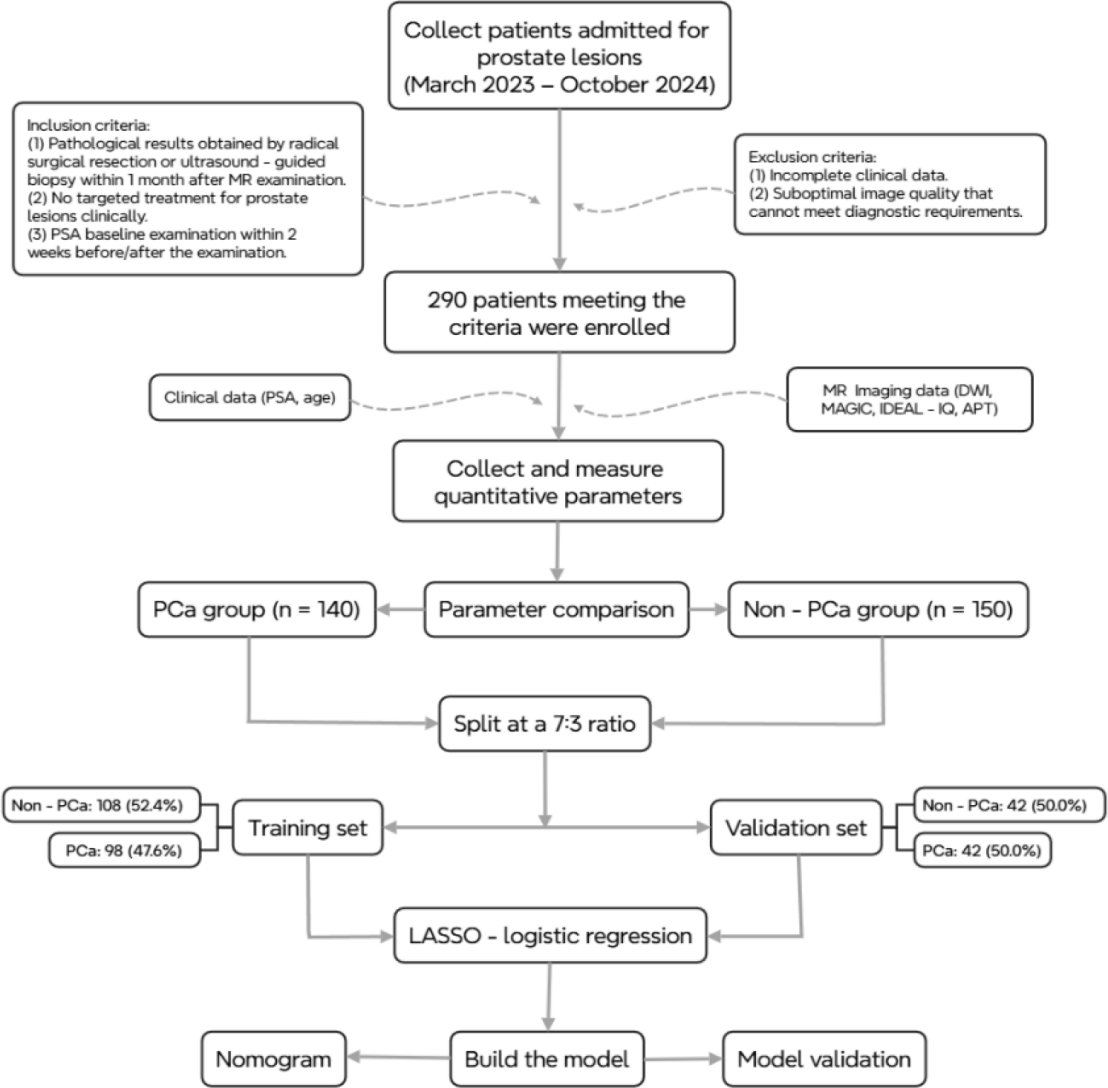
Flowchart.

The dataset was randomly split into a training set (70%) and a test set (30%), and internal validation was performed using stratified bootstrap resampling (B = 1000) to estimate and correct for optimism in the model’s performance. Evaluate the model using receiver operating characteristic curves (ROC), goodness-of-fit tests (Hosmer-Lemeshow), calibration curves, and clinical decision curve analysis (DCA), and present the model in the form of a nomogram. Visualized as a nomogram and Implemented via an online Shiny application (access: http://www.shinyapps.io/). The workflow is illustrated in **Figure 1**.

## 3 Results

### 3.1 Interobserver agreement test

Excellent reproducibility was observed for all quantitative parameters (**Table 2**), with ICC values as follows: 0.98 for ADC, 0.99 for T1, 0.96 for T2, 0.98 for PD, 0.97 for FF, 0.93 for R2*, and 0.88 for MTRasym(3.5ppm) (**Table 2**).

**Table 2:** Interobserver agreement test (ICC).

Parameter	ADC	T1	T2	PD	FF	R2*	MTRasym(3.5ppm)
ICC	0.98	0.99	0.96	0.98	0.97	0.93	0.88

ICC >0.75 = excellent; 0.75–0.50 = good; <0.50 = poor.

### 3.2 Demographics characteristics and QMRl parameters

In the Non−PCa group (n=150), 68 patients (45.3%) had prostatectomy specimens and 82 (54.7%) had biopsy specimens. In the PCa group (n=140), diagnoses were established exclusively on radical prostatectomy specimens, with complete Gleason scores recorded: Gleason 6, n=12 (8.6%); Gleason 7, n=45 (32.1%); Gleason 8, n=28 (20.0%); Gleason 9, n=33 (23.6%); and Gleason 10, n=22 (15.7%); Among the 140 PCa cases, 12 (8.6%) were indolent prostate cancer (Gleason 6) and 128 (91.4%) were clinically significant prostate cancer (Gleason ≥3 + 4); there were 208 radical prostatectomy specimens (71.7%) and 82 biopsy specimens (28.3%); as shown in **Table 3**.

**Table 3:** Distribution of pathology specimen sources and Gleason score distribution in the PCa group.

Group	Radical prostatectomy n (%)	Biopsy n (%)	Gleason n (%)				
			6	7	8	9	10
PCa (n=140)	140 (100.0%)	0 (0.0%)	12 (8.6%)	45 (32.1%)	28 (20.0%)	33 (23.6%)	22 (15.7%)
Non-PCa(n=150)	68 (45.3%)	82 (54.7%)	—	—	—	—	—
Total (n=290)	208 (71.7%)	82 (28.3%)	indolent 12(8.6%)	clinically significant 128(91.4%)			

1. ”—” indicates not applicable (no Gleason score available for the Non−PCa group). 2. Gleason scores in the PCa group are based on radical prostatectomy specimens.

Except for age, all other parameters showed significant differences between the PCa and non-PCa groups (*P* < 0.05 for all), as shown in **Table 4**.

**Table 4:** Comparison of baseline characteristics between PCa and non-PCa groups.

Parameter	non-PCa groups (n=150)	PCa groups (n=140)	Statistical values(*Z*/*t)*	*P*-value
ADC	0.92 (0.86, 1.00)	0.66 (0.54, 0.76)	*Z* = 12.719	<0.001
T2	94.34 (86.49, 102.08)	83.69 (79.07, 90.36)	*Z* = 7.000	<0.001
T1	1349.50 (1207.18, 1510.75)	1266.31 (1160.53, 1353.06)	*Z* = 4.508	<0.001
PD	78.394 ± 4.721	72.208 ± 5.285	*t* = 10.526	<0.001
APT	2.10 (1.84, 2.42)	2.81 (2.50, 3.16)	*Z* = 11.851	<0.001
FF	1.24 (0.33, 1.98)	1.81 (1.10, 2.49)	*Z* = 4.131	<0.001
R2*	20.905 ± 4.272	22.641 ± 4.682	*t* = 3.301	0.001
PSA	9.96 (5.01, 15.34)	57.23 (24.80, 98.63)	*Z* = 11.249	<0.001
age	67.50 (61.00, 72.00)	64.00 (59.00, 71.00)	*Z* = 1.396	0.163

The entire cohort was split into training and validation sets at a 7:3 ratio. All parameters showed no statistically significant intergroup differences (*P*>0.05 for all), indicating good consistency in data distribution between the two sets, as shown in **Table 5**.

**Table 5:** Comparison of baseline characteristics between training and validation sets.

Parameter	Training Sets (n=206)	Validation Sets (n=84)	Statistical values (*Z*/*t*/χ^2^*)*	*P*-value
ADC	0.83 (0.68, 0.93)	0.81 (0.64, 0.92)	*Z* = 0.280	0.78
T2	88.90 (81.00, 97.70)	87.59 (80.62, 96.42)	*Z* = 0.605	0.545
T1	1297.54 (1199.84, 1401.50)	1296.94 (1161.00, 1420.18)	*Z* = 0.372	0.710
PD	75.600 ± 5.992	74.935 ± 5.584	*t* = 0.875	0.382
APT	2.47 (2.09, 2.81)	2.50 (2.10, 2.84)	*Z* = 0.482	0.630
FF	1.51 (0.67, 2.26)	1.42 (0.50, 2.42)	*Z* = 0.120	0.904
R2*	21.608 ± 4.592	22.073 ± 4.457	*t* = 0.788	0.431
PSA	15.98 (7.75, 53.31)	16.20 (8.60, 59.25)	*Z* = 0.178	0.858
age	66.00 (59.00, 71.00)	67.00 (60.25, 72.25)	*Z* = 0.502	0.616
group (n,%)	non-PCa: 108 (52.4%)	non-PCa: 42 (50.0%)	χ^2^ = 0.141	0.708
	PCa: 98 (47.6%)	PCa: 42 (50.0%)		

### 3.3 Variable selection

#### 3.3.1 Variables selected via LASSO regression

**Figure 2:**
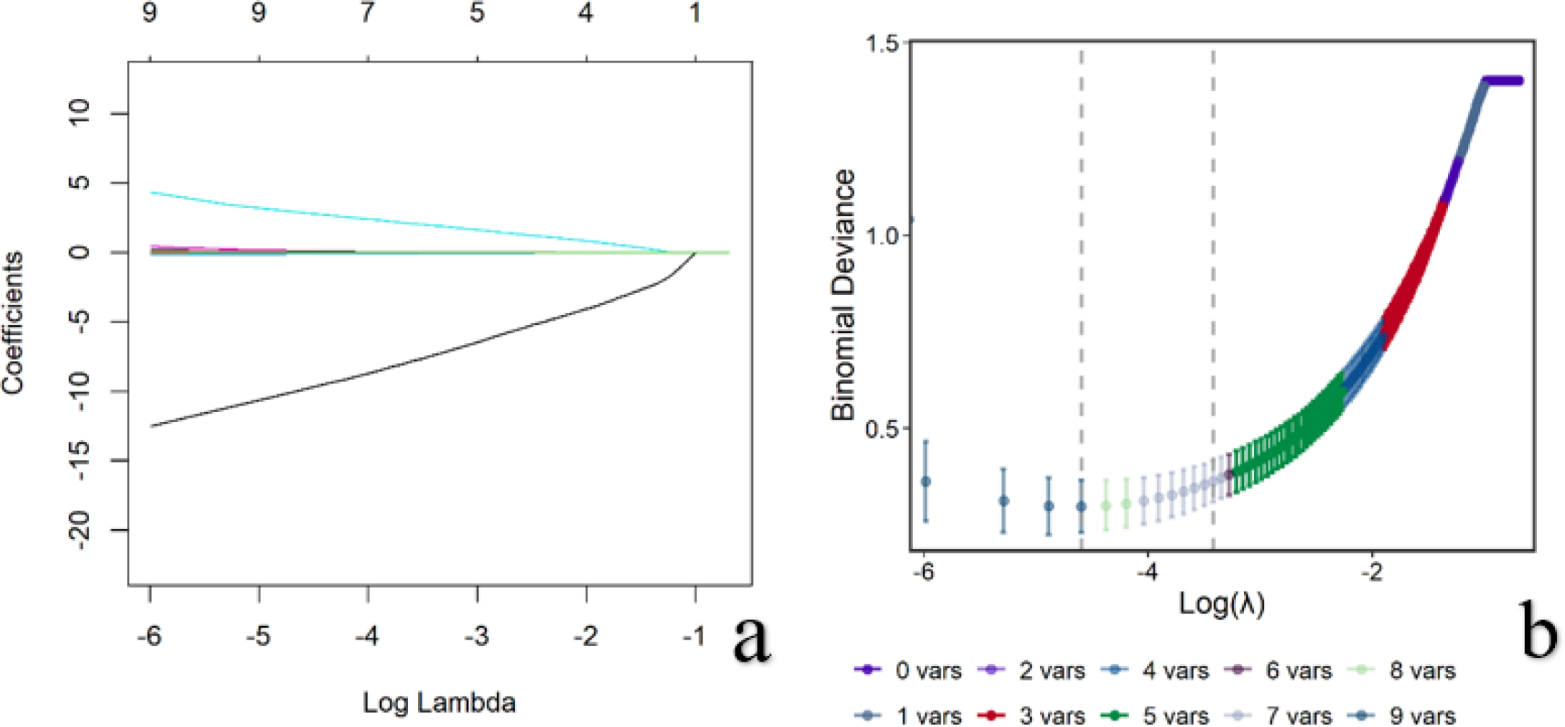
Results of LASSO regression for variable selection. [**(a)** LASSO coefficient path plot; **(b)** Cross-validation curve of LASSO regression].

LASSO regression was applied to screen the aforementioned nine parameters for selecting features predictive of PCa. The optimal λ parameter (Lambda.lse = 0.032) was determined by 5-fold cross-validation, where λ minimized the validation error. Regression coefficients with non-zero values under this λ identified seven PCa-predictive parameters: ADC, T2, T1, PD, ATP, R2^*^, and PSA. These results are illustrated in **Figure 2**.

#### 3.3.2 Logistic regression analysis results

Multivariable binary logistic regression analysis was performed with PCa diagnosis as the dependent variable and the seven LASSO-selected predictors as independent variables. The results demonstrated that ADC, T2, APT, and PSA were statistically significant independent predictors of PCa (all *P* < 0.05), as presented in **Table 6**.

**Table 6:** Multivariable binary logistic regression analysis of PCa predictors.

Predictor	Partial β	SE	*z-*score	OR (95% CI)	*P*-value
ADC	-18.131	6.384	2.84	0.002 (0.000~0.004)	0.005
T2	-0.145	0.062	2.333	0.865 (0.766~0.977)	0.02
T1	0	0.004	0.005	1.000 (0.993~1.007)	0.996
PD	-0.351	0.188	1.867	0.704 (0.487~1.018)	0.062
APT	7.954	3.114	2.554	2846.062 (6.358~1274074.294)	0.011
R2	0.312	0.189	1.65	1.366 (0.943~1.979)	0.099
PSA	0.19	0.078	2.449	1.209 (1.039~1.408)	0.014

### 3.4 Development and internal validation of the PCa risk prediction model

#### 3.4.1 Development of the predictive model

**Figure 3:**
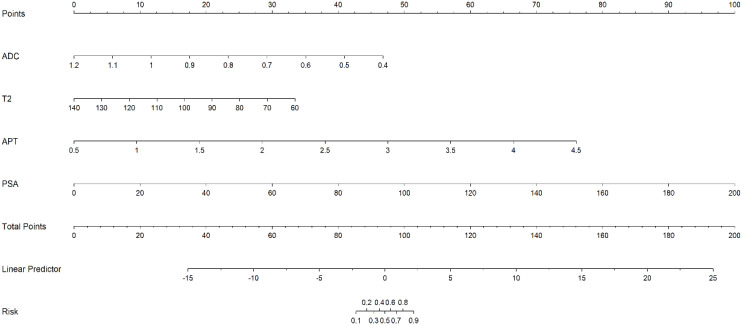
Point-of-care tool for MRI-guided prostate cancer risk assessment. [A nomogram integrating multiparametric MRI (ADC, T2, APT) and serum PSA to estimate individualized PCa probability at initial clinical encounter].

A PCa risk prediction nomogram (**Figure 3**) integrating four QMRI biomarkers (ADC, T2, APT) and serum PSA was constructed from the logistic model. Clinicians may input patient-specific measurements to estimate PCa probability.

**Figure 4:**
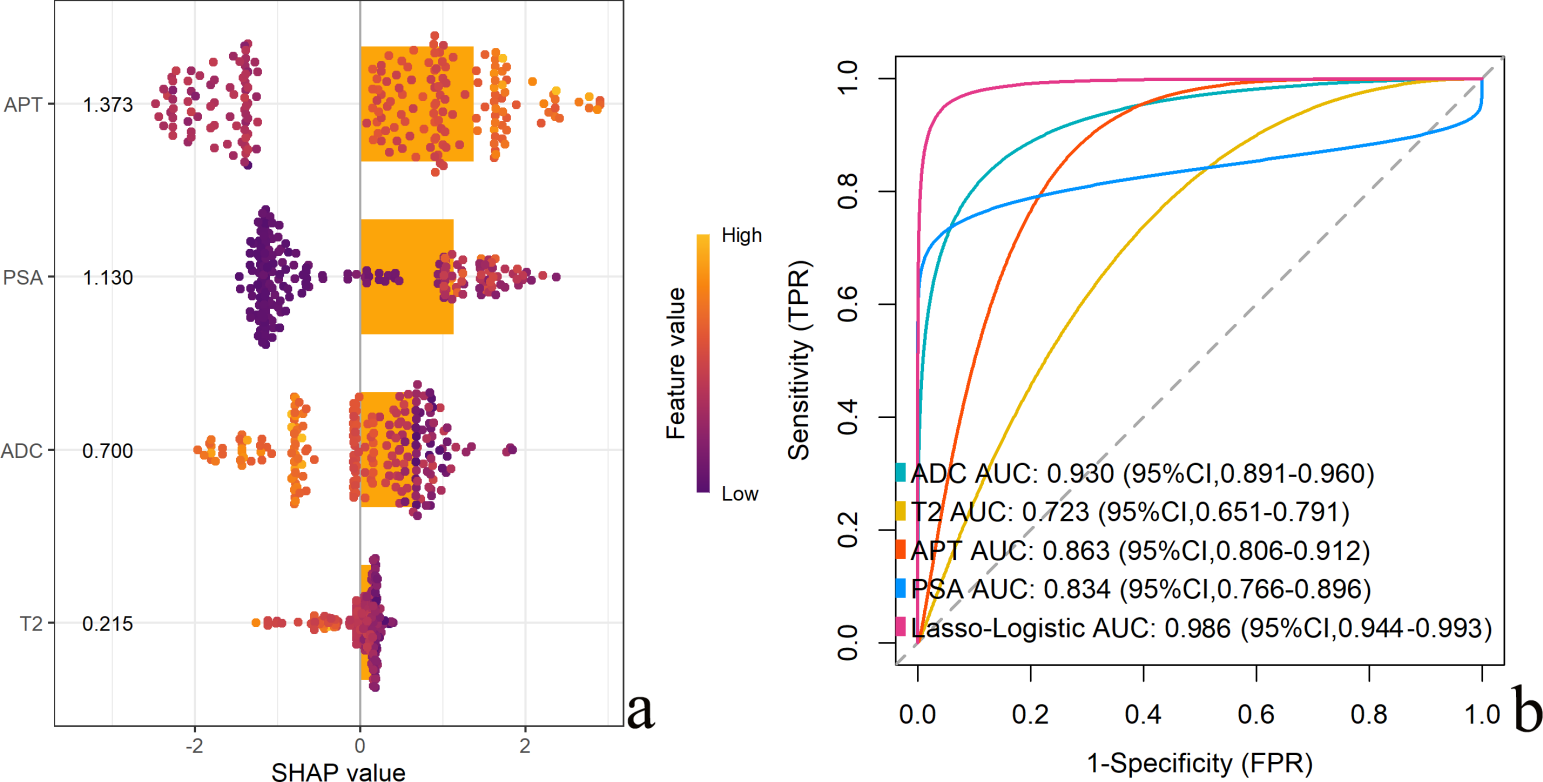
Predictor contributions and diagnostic performance of integrated model versus univariate predictors. [**(a)** SHAP summary plot ranking predictor contributions; **(b)** ROC curves comparing model and univariate diagnostic performance].

As demonstrated in **Figure 4** by SHAP analysis, the contribution values of predictors in descending order were APT, PSA, ADC, and T2. The predictive model performance curve revealed an AUC of 0.986, statistically superior to any single-parameter prediction.

#### 3.4.2 Model validation

**Figure 5:**
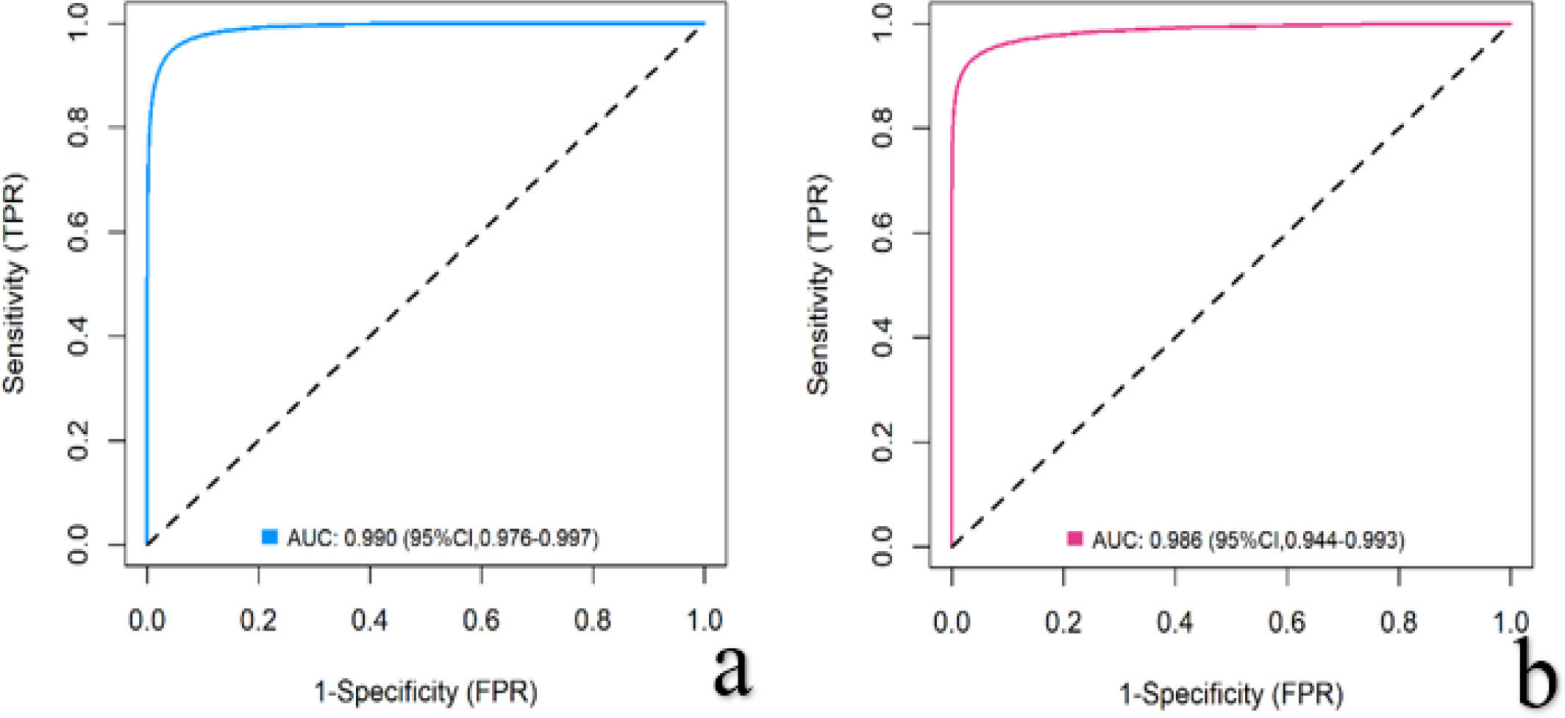
ROC curves demonstrating model performance. [**(a)** Training cohort; **(b)** Independent validation cohort].

Internal validation confirmed the model’s robust performance in discriminating PCa from non-PCa. The model demonstrated excellent discrimination, with AUCs of 0.990 (95% CI: 0.976-0.997) in the training set and 0.986 (95% CI: 0.944-0.993) in the validation set (**Figure 5**), alongside high sensitivity (97.0% and 92.3%) and specificity (95.2% and 93.3%). Goodness-of-fit tests showed no significant deviation in either set (P = 0.122 and P = 0.787).

**Figure 6:**
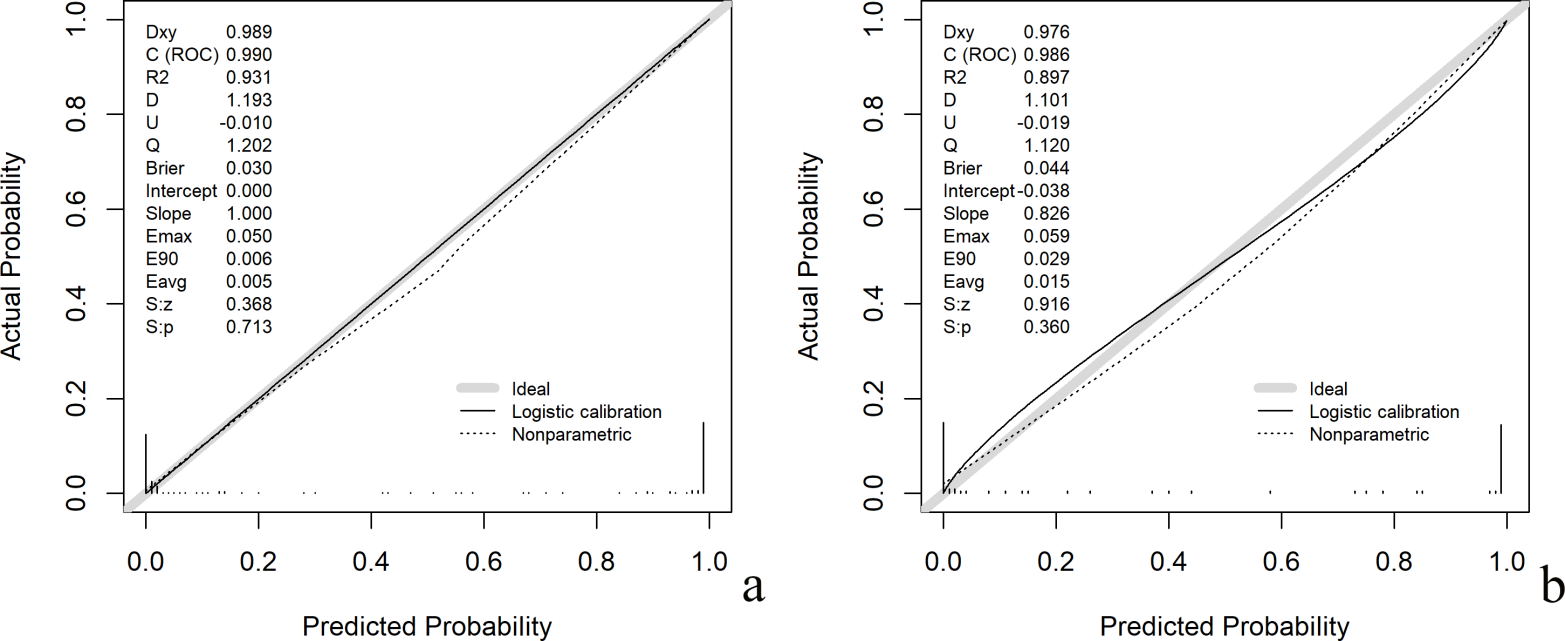
Calibration accuracy across development and validation cohorts. [**(a)** Training cohort; **(b)** Validation cohort].

Calibration plots showed that, in the training set, the model had a Brier score of 0.030, a calibration intercept of 0.000, and a calibration slope of 1.000, indicating excellent calibration in the development sample. In the internal validation set the Brier score increased to 0.044, the intercept was −0.038, and the slope decreased to 0.826. Although discrimination (AUC) remained high, the calibration slope < 1 in the validation set indicates modest overfitting, with predicted probabilities too extreme (high risks overestimated and low risks underestimated) (**Figure 6**).

**Figure 7:**
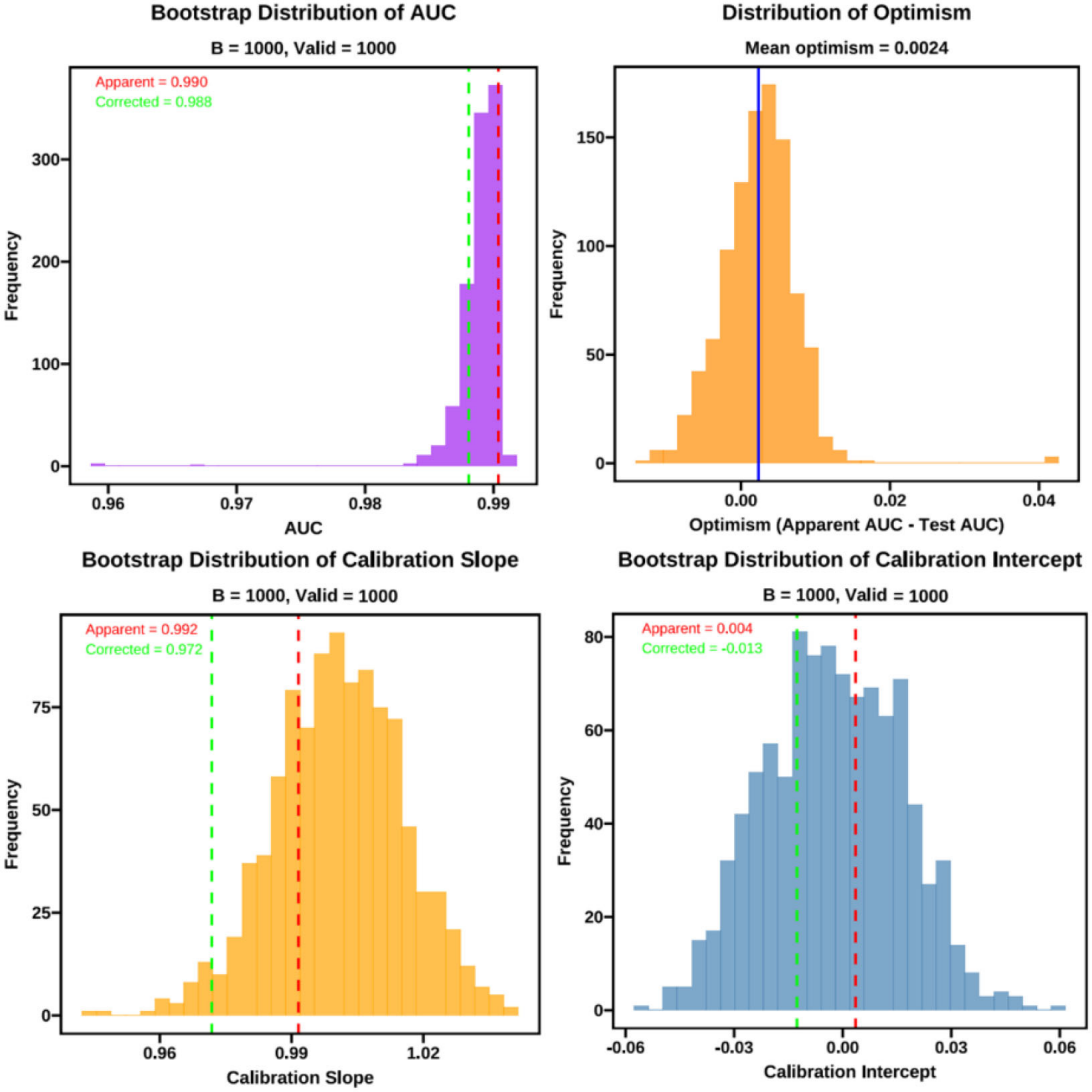
Bootstrap-related parameters.

To further assess model stability and to correct for optimism, we performed bootstrap validation with B = 1000 replications. The mean apparent AUC was 0.991, the mean test AUC (bootstrap models applied to the original sample) was 0.989, yielding a mean optimism of 0.002 and an optimism−corrected AUC of 0.988 (percentile bootstrap 95% CI: 0.986–0.991; **Table 7**). Calibration assessment showed an apparent intercept of 0.004 and an optimism−corrected intercept of −0.013 (95% CI −0.038 to 0.031); the apparent calibration slope was 0.992 and the optimism−corrected slope was 0.972 (95% CI 0.971–1.029) (**Table 8**). Bootstrap−related parameters are summarized in **Figure 7**.

**Table 7:** AUC performance (bootstrap internal validation, B = 1000).

Metric	Apparent (P_app)	Mean P_boot	Mean P_test	Mean optimism (P_boot − P_test)	Optimism-corrected (P_app − O)	95% CI (percentile)
AUC	0.990	0.991	0.989	0.002	0.988	(0.986, 0.991)

**Table 8:** Calibration metrics (bootstrap internal validation, B = 1000).

Calibration metric	Apparent (Estimate)	Mean_boot	Mean_test	Mean optimism	Optimism-corrected	95% CI (percentile)
Intercept	0.004	−0.001	−0.003	0.001	−0.013	(−0.038, 0.031)
Slope	0.992	1.003	1.001	0.002	0.972	(0.971, 1.029)

Apparent refers to the apparent estimate computed on the full sample. Mean_boot is the average estimate across the bootstrap samples (performance measured within each bootstrap sample). Mean_test is the average performance of the bootstrap−fitted models when applied to the original sample. Mean optimism = Mean_boot − Mean_test. Optimism−corrected = Apparent − Mean optimism. Ninety−five percent confidence intervals were derived using the percentile bootstrap method (2.5%–97.5%). Internal validation was performed using stratified bootstrap resampling (B = 1000, stratified by outcome).

#### 3.4.3 Clinical applicability analysis

**Figure 8:**
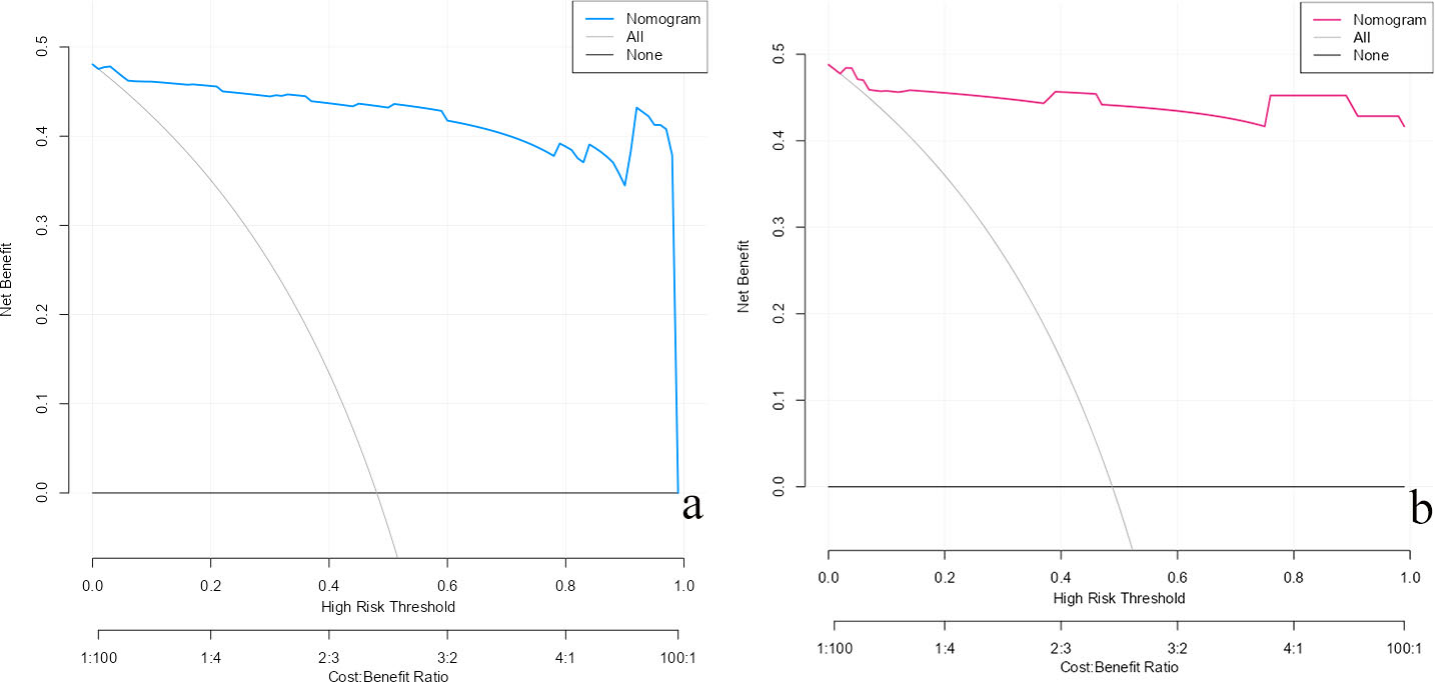
Decision curve analysis (DCA) of the predictive model. [**(a)** Training set; **(b)** Validation set].

The DCA was conducted using the nomogram-derived risk predictions as the test variable and PCa diagnosis as the state variable. The analysis demonstrated that the nomogram provided a superior net benefit across a wide range of threshold probabilities compared to the ‘treat-all’ and ‘treat-none’ strategies (**Figure 8**), underscoring its robust clinical utility.

### 3.5 Development of a web-based clinical decision support application

To facilitate streamlined access to the prediction model, we developed an innovative web-based risk calculator (https://zyf-prostate.shinyapps.io/dynnomapp/) that provides individualized probability estimates of PCa prior to biopsy.

## 4 Discussion

Prostate cancer is a common urogenital malignancy in older men. While the 5-year survival rate for asymptomatic or early-stage patients is 100% (16), this highlights the critical importance of standardized screening for the detection of clinically significant disease. Although mp-MRI has standardized prostate imaging protocols, its interpretations remain subjective (17). QMR transcends conventional imaging by delivering quantifiable biomarkers that minimize diagnostic variability and reveal early microstructural alterations undetectable by traditional MRI (5). We integrated multiparametric QMRI (DWI, MAGiC, IDEAL-IQ, APT) with clinical data and identified ADC, T2, MTRasym(3.5ppm), and PSA as independent predictors through multivariable modeling. The prediction model constructed with these factors achieved an AUC of 0.986 (95% CI: 0.944–0.993), with a sensitivity of 92.3% and a specificity of 93.3%, the optimism−adjusted area under the curve was 0.988 (bootstrap 95 % CI: 0.986–0.991). Goodness-of-fit tests and calibration curves indicated satisfactory calibration and predictive consistency of the model. DCA demonstrated favorable clinical applicability.

### 4.1 Multiparametric QMRI

Quantified through DWI, the ADC objectively evaluates water molecule diffusion by monitoring intra-tissue Brownian motion. Pathophysiologically, alterations in ADC reflect microstructural changes during carcinogenesis, thereby providing both qualitative and quantitative assessments for PCa (18). The characteristic decrease in ADC, reflecting restricted water diffusion, is consistently observed during prostate cancer (PCa) progression and is indicative of higher malignancy (19). Similarly, Studies indicate that lower ADC values, which are associated with higher tumor cellularity and poorer differentiation, are further associated with higher Gleason/ISUP grades and an increased risk of extracapsular extension (20). Lu et al. have shown that DWI and its ADC quantitative analysis can immediately improve differential diagnostic efficacy in routine clinical practice (21). Shao et al. (22) found that constructing a predictive model using T2WI, DWI, and ADC can predict tumor aggressiveness information that can only be obtained through pathological evaluation, thereby assisting clinicians in diagnosing prostate cancer, clinically significant prostate cancer, and pathological grading. In this study, there was a statistically significant difference in ADC (b=1500 s/mm²) between the PCa group and the non-PCa group (P<0.001), and ADC was an independent predictor of PCa. Consequently, a reduction in ADC can serve as a key imaging biomarker for as a key imaging biomarker for the diagnosis of PCa.

Evidence shows that MAGiC not only provides an efficient ‘one-stop’ scanning solution, but its quantitative T2 values also serve as a stable and reliable diagnostic biomarker, demonstrating clear application potential in improving the specificity of prostate cancer diagnosis and assisting in risk stratification. T2 values are derived from the MAGiC technique, which can objectively quantify the differences between tumor tissue and normal tissue caused by changes in the water molecule environment within the tissue due to increased tumor cell density and destruction of glandular structure, thereby reducing errors from subjective judgment (23). PCa tissues typically exhibit significantly lower T2 values than normal peripheral zone tissues. Synthetic T2WI shows image quality comparable to conventional T2WI, which will support the clinical implementation of MAGiC parameters for prostate MRI assessment (24). Kang KM, et al. have confirmed that the T2 values and ADC values obtained by MAGiC are negatively correlated with the Gleason score. In terms of diagnosing clinically significant prostate cancer, quantitative T2 maps exhibit diagnostic performance comparable to the apparent diffusion coefficient, and combining both can further enhance diagnostic efficacy (24). cheng et al. evaluated the diagnostic efficacy of PCa based on the SyMRI quantitative relaxation parameter model and found that its diagnostic efficacy was equivalent to DWI, while the combined model was significantly better than prostate imaging Reporting and Data System (25). This study corroborates these findings: statistically significant T2 differences between PCa and non-PCA groups confirm T2’s diagnostic value. Although T2 values have a certain degree of effectiveness in the diagnosis of prostate cancer, some studies believe that ADC values are more advantageous in terms of diagnosis and assessing the aggressiveness of prostate cancer (26, 27). This study indicate that quantitative T2 values can serve as independent predictors for PCa, marking a step forward in the precise diagnosis of prostate cancer. Of course, this requires further research in the future to explore and validate.

APT imaging is a molecular MRI technique based on chemical exchange saturation transfer (CEST), which quantifies the exchange kinetics between amide protons (-NH) in mobile proteins/polypeptide chains and water protons (-OH). It can serve as a biomarker for dynamic changes in intracellular protein/polypeptide content (15). Its application in oncology leverages the hyperproliferative phenotype and enhanced protein synthesis characteristic of malignancies, showing growing promise in tumor detection and treatment response assessment (28). PCa exemplifies these traits: increased cellular density elevates structural protein content, while upregulated secretion of macromolecules accelerates proton exchange efficiency – collectively manifesting as elevated APT signals in tumor foci (29) Concurrently, metabolically driven hypoxia induces tissue acidosis (pH 6.3 ± 0.4 in PCa vs. 7.0 ± 0.2 in normal tissue), which modulates amide proton exchange rates through pH-dependent chemical exchange CEST effects (30). Reesink etal. utilized CEST to detect APT as well as amine and/or creatine levels in the prostate in a non-invasive manner (31). Takayama et al. explored the application value of APT imaging in evaluating the Gleason score (GS) of prostate cancer and found that the APT index of prostate cancer patients with a GS of 7 was higher than that of other GS groups (32). Fang et al. explored the value of APT in evaluating the invasive risk of PI-RADS v2.13–5 lesions and found that APT could be used as an important biomarker for PCa risk stratification, and the combination of APT with PSA and ADC had the highest diagnostic efficiency (33). Zhou et al. summarized the application of APT in PCa and found that APT imaging could provide higher value than ADC value in differentiating transitional zone PCa from benign prostatic hyperplasia and interstitial prostatic hyperplasia (34). Our data also validate this synergy: significant intergroup differences in the magnetization transfer ratio asymmetry confirm APT’s unique capacity to enhance diagnostic accuracy by probing tumor metabolism. Current evidence indicates that it cannot only effectively detect tumors, but more importantly, has great potential in distinguishing clinically significant invasive cancers. Integrating it into existing multi-parametric MRI examinations is expected to significantly improve the performance of diagnostic models without substantially increasing scan time, making it one of the important directions for future prostate imaging development.

### 4.2 Existing PCa prediction models

Existing predictive models for prostate cancer predominantly utilize radiomic features, clinical parameters (PSA), PI-RADS assessments, and genetic biomarkers. Wang et al. pioneered a multimodal MRI radiomics framework integrating DWI, DCE and T2−weighted features with clinical variables (PSA, free PSA and digital rectal examination) for risk stratification; this combined model achieved an AUC of 0.898 and significantly outperformed a model based on clinical variables alone (38). Building on this approach, Chen et al. developed a multiparametric MRI radiomics nomogram that incorporates PI−RADS scoring together with clinical factors and reported outstanding discriminatory performance for distinguishing prostate cancer from benign lesions (AUC = 0.980) (39).

Regarding serum markers, disruption of the prostate–blood barrier by inflammation, benign disease or malignancy elevates circulating PSA, which underlies PSA’s ongoing utility in diagnostic stratification and treatment monitoring (36). Roscigno M et al. further demonstrated that PSA density (PSAD) is significantly associated with the risk of pathological upgrading and that combining PSAD with mpMRI in active−surveillance cohorts can reduce futile biopsies by approximately 77.5% (35). Consistent with these findings, our cohort showed significantly higher PSA levels in the PCa group than in the non−PCa group, supporting the role of PSA as an evidence−based adjunct to multiparametric MRI in diagnostic workflows.

Molecular diagnostics have likewise advanced prostate cancer detection. Pepe et al. evaluated the urine−based SelectMDx assay (measuring expression of HOXC6, DLX1 and related markers) and showed that it can predict high−risk disease and help avoid repeat biopsies in low−risk men (37). They also explored optimal combinations of PCA3 and free/total PSA to refine biopsy decision−making and substantially reduce over−diagnosis (40). In addition, 68Ga−PSMA PET/CT, owing to its high specificity, offers complementary “subtractive” and “additive” strategies for clinically significant prostate cancer (csPCa): it can exclude low−risk cases to avoid unnecessary biopsies and enhance targeting accuracy for biopsy when performed (41). Collectively, these noninvasive or minimally invasive approaches—radiomics, PSA−derived metrics, molecular assays and PSMA PET—create complementary diagnostic pathways that reduce unnecessary biopsies and improve diagnostic accuracy.

QMRI offers direct histopathological and physiological information (e.g., indicators of cellular density and metabolic activity), which supplements clinical measures and standard imaging findings and may improve disease characterization. To date, no study has combined multiparametric quantitative MRI metrics with clinical variables to predict prostate cancer. Therefore, we sought to develop a prostate cancer prediction model based on independently selected predictors from multivariable screening.

We developed the first QMRI–clinical prostate cancer prediction model (QMR−CPM) that integrates QMRI biomarkers (T2, ADC, APT) with the clinical parameter PSA. Using quantitative measurements, the model achieved near−perfect discrimination in the derivation cohort (AUC = 0.990; 95% CI, 0.976–0.997) and demonstrated excellent performance in the validation cohort (AUC = 0.986; 95% CI, 0.944–0.993; sensitivity =92.3%, specificity=93.3%). The optimism−corrected AUC was 0.988. Internal validation supports the model’s strong discriminatory ability and near−ideal calibration in this cohort, and decision curve analysis indicates favorable clinical utility.

### 4.3 Limitations

This study has several important limitations. First, according to the TRIPOD framework, it represents model development with same−source internal validation (Type 2b) and has not undergone independent external, multicenter validation. Therefore, prior to clinical implementation the model must be validated across different institutions, scanner vendors, field strengths, and patient populations to confirm its generalizability. Second, the pathological reference standard comprised both biopsy and radical prostatectomy specimens; some cases were characterized only by biopsy, which carries a risk of undergrading. Moreover, a small number of indolent (Gleason 6) prostate cancer cases were present in the cohort, which may affect model training and performance estimates. Third, PI−RADS scores were not consistently documented across the study cohort, so a head−to−head comparison between the quantitative model and PI−RADS (or combined clinical–PI−RADS strategies) was not possible and should be addressed in future work. Fourth, the quantitative MRI metrics used here are sensitive to acquisition protocol, vendor and scanner, creating potential device−dependent bias; future studies should adopt isotropic 3D acquisitions, harmonize protocols across vendors (for example through phantom calibration or standardized sequences), and perform multicenter scanning to evaluate parameter robustness and reduce scanner dependency. The precision of the APT coefficient estimate may be improved in future studies with larger sample sizes and standardized acquisition protocols. Given these limitations, we advise caution in applying the model in routine clinical practice until external validation and, where appropriate, recalibration or shrinkage have been completed.

## 5 Conclusion

In conclusion, this study successfully developed and validated a novel prediction model for PCa by integrating multimodal QMRI parameters with clinical data. The model, which incorporates ADC, T2, MTRasym(3.5ppm), and PSA, demonstrated exceptional discriminative performance, robust calibration, and compelling clinical utility. This tool provides a highly objective, reproducible, and non-invasive aid for the clinical diagnosis and risk stratification of prostate lesions. Although the model demonstrated good internal performance in our cohort, owing to its limitations it requires external validation in independent multicenter cohorts — and recalibration if necessary — prior to clinical application.

## Data Availability

The raw data supporting the conclusions of this article will be made available by the authors, without undue reservation.
